# Multi-year survey data on public opinion on driverless vehicles in Australia and New Zealand

**DOI:** 10.1016/j.dib.2025.111273

**Published:** 2025-01-10

**Authors:** Amolika Sinha, Trevor Wang, Macgregor Buckley, Rita Excell, Rahila David

**Affiliations:** a Australian Road Research Board (ARRB), National Transport Research Board, Australia; b Centre for Connected and Automated Transport, Australia and New Zealand; c The Australia and New Zealand Driverless Vehicle Initiative (ADVI), ARRB, Australia

**Keywords:** Public opinion, Driverless vehicles, COVID-19 impact, Connected automated vehicles, Consumer acceptance, Multi-year survey

## Abstract

Highly automated driving (HAD) vehicles are predicted to have several societal benefits, including reduced accidents and fatalities, reduced congestion, efficient traffic networks and improved productivity. However, the adoption of technology is dependent on the willingness of people to adopt the technology. For a smooth transition to driverless vehicles, it is essential to investigate the public perception of driverless vehicles. This paper presents the multi-year survey data on public opinion on driverless vehicles in Australia and New Zealand. The survey has four rounds undertaken over a period of six years that can provide insights about the sentiment of Australians and New Zealanders over time. The data presented in the paper can be used in research studies that can benefit vehicle manufacturers, policy makers and researchers responsible for and interested in the safe and successful deployment of automated and driverless vehicles on Australian and New-Zealand roads in the future. Additionally, the last round of the survey also consists of Covid-19 questions and can be used to investigate Covid-19′s effect on mobility.

Specifications Table

**Table:** 

Subject	Applied Psychology
Specific subject area	Public Opinion on Driverless Vehicles in Australia and New Zealand
Data format	Raw, Filtered
Type of data	Table, Chart, Graph, Figure
Data collection	A 20-min survey was distributed by Qualtrics to the participants that were over 18 years old.
Data source location	Australia • New Zealand
Data accessibility	Repository name: Mendeley Data identification number: 10.17632/9333msxhrc.1 Direct URL to data: https://data.mendeley.com/datasets/fzmpmcnn3y/1

## 1 Value of the Data

• The survey data represents the inaugural study of its kind conducted within the Australian context.
• The survey is comprised of an extensive dataset of 6000 individual entries.
• It stands as a robust multi-year survey, extending over several years.
• This survey encompasses the period marked by the COVID-19 pandemic, thereby providing invaluable insights into the shifts in public opinion attributable to the pandemic's influence.
• This temporal span offers a unique opportunity to discern the nuanced changes in societal attitudes precipitated by the unprecedented circumstances of the COVID-19 era.

## 2 Background

The Volpe National Transportation Systems Centre's overview of research on the factors affecting consumer adoption of innovative technologies has revealed that interest in highly automated driving (HAD) vehicles is mixed and leans negative based on the surveys reviewed (Hassol et al.) [1]. There are numerous studies indicating the benefits of HAD vehicles [2,3] including improving community-based trip sharing [4], providing several public and private benefits [5], building a sustainable transport system [6] and eliminating the human errors that contribute to 90 % of collisions [7]. Despite the benefits that HAD promises, many people remain sceptical about adoption of the technology. Multiple studies have attempted to investigate public perception [8] and trust in the technology [9]. Studies indicate that the acceptance of HAD vehicles is dependent on multiple determinants [10]. Similar to the study done by Hassol et al. [1] in 2019, another study [11] done on university populations, found that the majority of US drivers are unwilling to ride in HAD vehicles. Therefore, it can be anticipated that a significant barrier to the diffusion of HAD vehicles originate from low public acceptance [[12], [13], [14]] and that the public's fear presents potential concern [15].

While there have been multiple review studies investigating public acceptance and intention to use HAD vehicle technology [[16], [17], [18], [19], [20], [21], [22], [23], [24], [25], [26], [27]] a knowledge gap remains [28]. Table 2 presents the previous studies that attempted to elicit the perceptions of HAD vehicles in the Australian and/or New Zealand demographics.

## 3 Data Description

This section discusses the organisation of the survey data in the repository. Table 1 presents the data description and Table 1 outlines the structure of the repository. There are four folders in the repository corresponding to each round of survey. Each folder contains two files – ADVI (The Australia and New Zealand Driverless Vehicle Initiative) questions and ADVI Public Acceptance Survey. The “ADVI questions” file presents the list of all the questions in the survey. The “ADVI Public Acceptance Survey” presents the responses collected for each question in the survey. It is noted that Some respondents did not finish the survey and hence some incomplete responses can be found in the survey. The column name represents the questions, and each row represents the responses of an individual participant.

**Table 1:** Structure of repository.

S No.	Folder name	File Name	Details	Path
1.	Round 1	ADVI #1 questions	List of question for survey 1	/Round 1/
2.		ADVI Public Acceptance Survey #1	Survey 1 Responses (Questions as column headings)	/Round 1/
3.	Round 2	ADVI #2 questions	List of question for survey 2	/Round 2/
4.		ADVI Public Acceptance Survey #2	Survey 2 Responses (Questions as column headings)	/Round 2/
5.	Round 3	ADVI #3 questions	List of question for survey 3	/Round 3/
6.		ADVI Public Acceptance Survey #3	Survey 3 Responses (Questions as column headings)	/Round 3/
7.	Round 4	ADVI #4 questions	List of question for survey 4	/Round 4/
8.		ADVI Public Acceptance Survey #4	Survey 4 Responses (Questions as column headings)	/Round 4/

## 4 Experimental Design, Materials and Methods

This section describes the method for the collection of the data.

### 4.1 Recruitment

A 20-minute survey was distributed by Qualtrics to the participants in Australia and New Zealand. The participants for the study were carefully chosen from both Australia and New Zealand. The selection process was designed to ensure that the distribution of participants accurately represents the demographic characteristics of the populations in both countries.

The research study recruited people who met the following criteria:

- Of at least 18 years of age; and
- Currently residing in Australia or New Zealand.

### 4.2 Questionnaire

A thorough survey (around 70+ items) was developed by the ADVI Survey Working Group, comprised of experts from academia, government, and industry. The questions in the survey were drawn in part from previous surveys reported in the international scientific literature, in part by the authors, and in part by feedback from ADVI partners from different sectors (academia, industry and government). The overarching aim was to seek public feedback on the following key issues:

• The level of awareness of HAD vehicle technology (e.g., whether individuals have heard about or seen a vehicle which can stay within the lane by itself), given exposure to technology and media [29] influences perceptions of technology.
• Sources, and degree, of concern regarding HAD vehicle-related issues (e.g., cyber security).
• In what driving scenarios and conditions drivers would be most likely to use HAD vehicles (e.g., when traffic is congested, when the driver is tired or fatigued, etc.)
• What activities drivers would undertake when driving is fully supported by automation (e.g., reading, interacting with other passengers, etc.)
• Opinions regarding HAD vehicles (e.g., are HAD vehicles safer than human drivers?)
• Willingness to pay for an HAD vehicle.

A previous study [30] found that parents projected the benefits of HAD vehicles to be advancing mobility and safety. However, several factors (parent's sex, residence area, child's age, etc.) were found to be crucial in HAD vehicle acceptance. Therefore, the survey includes questions eliciting parents’ views on allowing their children to use HAD vehicles. Additionally, some studies have found that men are more likely to adopt self-driving cars than women [31,32]. Henceforth, the survey includes questions on sociodemographic information.

Once developed, every round of the survey was distributed to 5000+ people across Australia and New Zealand through the online survey platform, Qualtrics. For each round of the survey, it is conceivable that a different set of individuals participated. Although there may have been instances where certain individuals took part in multiple survey rounds, this overlap was not intentional nor guaranteed. This translates that this study did not aim to track the evolving opinions of specific individuals over time.

Table 2 presents the date of first and last response recorded and the number of participants.

**Table 2:** Survey sample information.

Survey Rounds	Date (First Response)	Date (Last Response)	Participants (after filtering)
Round 1 (2016)	29/08/2016	11/09/2016	Aus - 4754
NZ- 0			
Round 2 (2017)	17/10/2017	8/11/2017	Aus - 4921
NZ - 1028			
Round 3 (2019)	08/09/2019	29/10/2019	AUS - 5295
NZ - 1082			
Round 4 (2021)	28/03/2021	14/04/2021	Aus - 5058
NZ - 1066			

For each round of the survey, it is conceivable that a different set of individuals participated. Although there may have been instances where certain individuals took part in multiple survey rounds, this overlap was not intentional nor guaranteed. This translates that this study did not aim to track the evolving opinions of specific individuals over time.

### 4.3 Survey design and process

Since the study involved human subjects, the survey methods (for each round) were approved by the University of New South Wales Human Research Ethics Committee (UNSW-HREC). Furthermore, any modifications in the research were regularly communicated with UNSW-HREC. Any progress was made in compliance in UNSW-HREC. A comprehensive questionnaire was developed as discussed in the previous “Questionnaire” section of this paper. A pilot testing (for each round) was done on 20 people which facilitated the refinement of questionnaire for comprehensibility. After the pilot testing, the survey was sent to participants by Qualtrics, a survey respondent recruitment and administration company. All participants were volunteers, who had elected to be on-call for surveys administered by Qualtrics. An information sheet about the research was provided to all participants at the start of the survey, and participants clicked a web link to indicate consent for the response collection. All the consenting participants were instructed to respond to an online survey. The order-of-presentation of the questionnaires was randomized and took approximately 20 min to complete. All the participants were paid a small amount by Qualtrics for their participation. The information provided by Qualtrics was in the form of anonymised responses to each of the survey questions. Further filtering was done to remove personal information such as geolocation, Recipient Last Name, Recipient First Name, Recipient Email etc. Further details can be found in the “Technical Validation” section of this paper below. Each round of the survey cost over $100,000, and overall, the four rounds of the survey cost around $500,000.

As well as questions related to HAD vehicles, the survey also collected sociodemographic information about participants including age, gender, employment status, educational attainment, monthly household income, the nature of the residence, and car ownership.

## Limitations

The research team has executed a comprehensive data cleansing process to ensure the integrity and accuracy of the results. It is a well-acknowledged fact within the research community that large datasets, particularly those amassed from high-volume sampling, are susceptible to the inclusion of aberrant responses that defy logical interpretation. Therefore, it is strongly recommended that researchers undertake rigorous sanity checks to mitigate such anomalies. Furthermore, it is important to note that during the data collection phase, there were modifications in the phrasing and format of certain survey questions. These alterations, while perhaps necessary, have imposed constraints on the multi-year analysis with respect to some of the questions, thus limiting the continuity of data comparison over the extended timeframe of the study. For example, in the measurement of willingness to pay, respondents in 2016 and 2017 only reported "same," "more," "a lot more," and vacancy values. However, in 2019 and 2021, additional data was collected for "less" and "a lot less". This change provided respondents with more precise options, improving insights into their preferences and ensuring their views were accurately represented. However, this change disrupted the continuity of data comparison across the years.

## Ethics Statement

The authors have adhered to the ethical standards as delineated in the "Data in Brief's Guide for Authors." Additionally, the requisite approval for conducting human research has been secured from the Human Research Ethics Committees (HRECs) at the University of New South Wales. The authors have further affirmed that either informed consent was procured from all participants involved in the study, or that all participant data was completely anonymized. Moreover, adherence to the data redistribution policies set forth by the involved platform(s) has been ensured.

## Credit Author Statement

**Amolika Sinha:** Conceptualisation, Data validation, Software, Writing and Editing, Revision.

**Macgregor Buckley:** Data Curation, Data Validation, Software, Reviewing, Revision.

**Trevor Wang:** Data Curation, Data Validation, Software, Reviewing, Revision.

**Rita Excell:** Supervision, Resources, Reviewing.

**Rahila David:** Supervision, Resources, Reviewing.

All authors have read and agreed to this version of the manuscript.

## Data Availability

Mendeley DataHighly Automated and Driverless Vehicles Public Acceptance Survey for Australia and NewZealand (Original data).
